# HIF‐1α is necessary for activation and tumour‐promotion effect of cancer‐associated fibroblasts in lung cancer

**DOI:** 10.1111/jcmm.16556

**Published:** 2021-05-04

**Authors:** Yana Zhang, Yangyang Bian, Yuan Wang, Yuanyuan Wang, Xixi Duan, Yuning Han, Lijing Zhang, Fei Wang, Zhuoyu Gu, Zhihai Qin

**Affiliations:** ^1^ Medical Research Center The First Affiliated Hospital of Zhengzhou University Zhengzhou China; ^2^ Henan International Joint Laboratory of Tumor Immune Microenvironment Zhengzhou China; ^3^ General Hospital of Ningxia Medical University Ningxia China; ^4^ Key Laboratory of Protein and Peptide Pharmaceuticals CAS‐University of Tokyo Joint Laboratory of Structural Virology and Immunology Institute of Biophysics Chinese Academy of Sciences Beijing China

**Keywords:** CAFs, CCL5, fibroblasts, HIF‐1α, lung cancer

## Abstract

Cancer‐associated fibroblasts (CAFs) activation is crucial for the establishment of a tumour promoting microenvironment, but our understanding of CAFs activation is still limited. In this study, we found that hypoxia‐inducible factor‐1α (HIF‐1α) was highly expressed in CAFs of human lung cancer tissues and mouse spontaneous lung tumour. Accordingly, enhancing the expression of HIF‐1α in fibroblasts via hypoxia induced the conversion of normal fibroblasts into CAFs. HIF‐1α‐specific inhibitor or HIF‐1α knockout (KO) significantly attenuated CAFs activation, which was manifested by the decreased expression of COL1A2 and α‐SMA. In vivo, during tumour formation, the expression of Ki‐67 and proliferating cell nuclear antigen (PCNA) in the tumour tissue with HIF‐1α KO fibroblasts was significantly lower than that of normal fibroblasts. Moreover, HIF‐1α in fibroblasts could activate the *NF‐κB* signalling pathway and enhance a subsequent secretion of CCL5, thus promoting the tumour growth. In conclusion, our results suggest that HIF‐1α is essential for the activation and tumour‐promotion function of CAFs in lung cancer (LC). And targeting HIF‐1α expression on CAFs may be a promising strategy for LC therapy.

## 1 INTRODUCTION

Lung cancer (LC) is characterized by high morbidity and mortality among cancers worldwide. The latest research estimates that by 2020, the 5‐year survival rate of LC will be only 19%, second only to pancreatic cancer and liver cancer.^1^ Because of the rapid proliferation and early metastasis of LC cells, about 57% of the LC patients were diagnosed at late clinical stage and the 5‐year survival rate was low, only 5%.^2^ Therefore, it is of great significance to explore the specific mechanism of LC progression. Recent research has shown that tumour microenvironment (TME) plays a crucial role in the development of LC. TME includes a variety of cell types, such as tumour cells, CAFs, vascular endothelial cells, lymphatic endothelial cells and immune cells.^3^ As CAFs are the core elements of TME, the research of CAFs has been greatly expanded in recent years.

In tumour microenvironment, CAFs remodel the extracellular matrix composition and induce the infiltration of immune cells, which leads to the reprogramming of TME structure. These changes provide an appropriate environment for tumour growth.^4^ In these processes, the versatility and plasticity of CAFs will generate a large number of subgroups, which can promote or inhibit tumour progression and invasion, depending on the circumstances.^5^ Meanwhile, the various origins of CAFs also contribute to its biological heterogeneity.^3^ So defining the distinctive roles and functions of each CAFs sub‐population will be helpful for understanding CAFs as a whole. CAFs are mainly derived from the activation of fibroblasts in the tumour stromal tissues. Fibroblasts can be activated into CAFs by some soluble signalling molecules in TME, thus leading to phenotype changes and functional genes being differentially expressed, thereby regulating the development of tumour.^3^ However, it is unclear how to categorize CAFs into functional subtypes. It is known that fibroblasts can be activated by excessive biochemical activators from the TME such as transforming growth factor‐β (TGF‐β), sonic hedgehog (SHH), bone morphogenetic protein (BMP), platelet‐derived growth factor (PDGF), IL‐1 and IL‐6.^5^, ^6^, ^7^ Furthermore, tumour cell condition medium (CM) has been reported to activate the fibroblasts and increase ECM deposition.^8^ After the fibroblasts activation, some protein markers are up‐regulated, including α‐smooth muscle actin (α‐SMA/ACTA2), fibroblast‐activating protein (FAP), platelet‐derived growth factor receptor‐α (PDGFRα), fibroblast‐specific protein 1 (FSP1/S100A4), collagen type Ⅰ Alpha 2 (COL1A2) and Podoplanin (PDPN), which are recognized as representative markers of CAFs.^9^ These markers are lack of sensitivity and specificity, and there is no really highly specific CAFs marker. Therefore, it is necessary to find new molecular markers to identify the functional heterogeneity of CAFs, which is of great significance to the development of effective targeted therapy for LC. However, there are few studies on the activation and function of CAFs in LC.

The main function of lung is to absorb the O_2_ for the whole body. The oxygen content of lung is between 3.0 and 87.248 kPa. However, within NSCLC, the oxygen levels ranged from 0.0931 to 6.118 kPa. Hypoxia is an important pathognomonic feature of many malignant tumours including LC.^10^ With the rapid growth of LC cells, the oxygen consumption of tumour tissues increases, which further aggravates the hypoxia of tumour tissues. HIF‐1α is a pivotal transcription factor involved in many biological processes, such as tumour cell survival, angiogenesis, invasion and tumour therapy.^11^, ^12^, ^13^ It is reported that HIF‐1α has been involved in the process of tissue fibrosis and cancer progression.^14^ According to the literature, deletion of HIF‐1α in FAP positive fibroblasts accelerates the growth of breast cancer in transgenic mice.^15^ The loss of HIF‐1α in endothelial cells restrains the growth of spontaneous breast tumour.^16^ However, other studies suggest that HIF‐1α induces the metabolic reprogramming of CAFs and increases glycolysis, thereby promoting tumour growth in breast cancer.^17^ In pulmonary fibrosis, HIF‐1α/PDK1‐mediated glycolytic reprogramming can promote myofibroblast differentiation and fibrosis.^18^ Therefore, the function of HIF‐1α in CAFs is multifaceted and complex. Nevertheless, the role of HIF‐1α in CAFs of LC has not been studied to date. As hypoxia is a common phenomenon of LC, it is very important to study the exact effect of HIF‐1α on CAFs.

In the present study, we found that the HIF‐1α*‐*expressed fibroblasts promoted the growth of LC. Our results also showed that the expression of HIF‐1α was increased under the stimulation of hypoxia, TGF‐*β*1 and tumour cells CM, which were necessary for the activation of CAFs and LC growth. Finally, the mechanistic study revealed that HIF‐1α‐expressed fibroblasts accelerated the growth of LC through secreting CCL5.

## 2 MATERIALS AND METHODS

### 2.1 Tumour models

All mice were maintained under specific pathogen‐free conditions and conducted with approval from the First Affiliated Hospital of Zhengzhou University Ethics Committee. The mouse spontaneous LC model (*TetO‐EGFR^L858R^; CCSP‐rtTA*) was a gift of Professor Lin Xi from Tsing Hua University. To induce the formation of the lung tumours, the mice were treated as described previously.^19^

### 2.2 Cell isolation and cultivation

Mouse Lewis lung cancer (LLC) cells were obtained from Professor Li Yan from the Academy of Military Medical Sciences. Human embryonic lung fibroblasts (MRC‐5) and A549 were obtained from ATCC. Mouse embryonic fibroblast (MEF) cells were isolated from C57BL/6J wild‐type mice embryonic according to previous methods.^20^ Mouse CAFs (mCAFs) and mouse normal fibroblasts (mNFs) were isolated from mouse spontaneous LC model (*TetO‐EGFR^L858R^; CCSP‐rtTA*). Human CAFs and NFs were isolated from lung adenocarcinoma tissues and adjacent normal lung tissues, which were obtained from three patients. Those patients were diagnosed with lung adenocarcinoma between May 2018 and June 2019 at the First Affiliated Hospital of Zhengzhou University. The CAFs and NFs were isolated according to the previously published protocol and passaged for up to 5‐7 populations in the subsequent experiments and cultured at normoxia condition.^19^ All the primary cells and cell lines were cultured with high glucose (4.5 mg/mL) Dulbecco's modified eagle medium (DMEM) containing 10% foetal bovine serum and 1% Penicillin‐streptomycin. Cells were maintained at 37°C, 5% CO_2_ and 20% O_2_ expect the special description. Hypoxic cells (1% O_2_) were maintained in a modular incubator chamber (Themo 4141) at 37°C, 1%O_2_, 5% CO_2_ and 94% N_2_.

### 2.3 Materials and reagents

Cocl_2_ was obtained from Sigma‐Aldrich. Mouse CCL5 ELISA kit and Lipofectamine 3000 were purchased from Invitrogen. Recombinant Mouse TGF‐beta 1 Protein (TGF‐*β*1) was purchased from R&D Systems. KC7F2, TAK‐779, MG‐132 and SC75741 were purchased from MedChemExpress. The indicated abbreviation of this paper are shown in Table S2.

### 2.4 Western blot

Cell samples were lysed in RIPA Lysis Buffer with 10% PMSF, 20% protease and phosphatase inhibitors for 30 minutes on ice, and then centrifuged at 13201 g for 20 minutes. The protein concentrations were measured with BCA Protein Assay Kit. The samples were separated by 10% SDS‐PAGE and transferred onto the nitrocellulose membrane. Then membranes were incubated with primary antibodies overnight at 4°C. At the second day, they were incubated with corresponding horseradish‐peroxidase‐conjugated (HRP) secondary antibodies. ECL Western blot Kit (CWBIO, #CW00495) was used to detect those bands in a ChemiDoc MP Imaging System (Bio‐Rad). The primary antibodies were used as follows: anti‐α‐SMA (Abcam, #ab21027; diluted 1:1000), anti‐COL1A2 (Proteintech, #14695‐1‐AP; diluted 1:1000), anti‐HIF‐1α (Abcam, #ab179483; diluted 1:1000), anti‐FAP (Origene, #TA347770; diluted 1:1000), anti‐CCR5 (Abclonal, #A20261; diluted 1:1000) and anti‐GAPDH (Abclonal, #WH105923; diluted 1:5000).

### 2.5 CRISPR Cas9 assay

Immortalized mouse embryonic fibroblast (iMEF) cells were a gift of Professor Lin Xi from Tsing Hua University. CRISPR‐Cas9 plasmids of HIF‐1α were purchased from Addgene (NCBI Reference Sequence: NM_001313919.1). The plasmids were transfected into 293T cells using Lipofectamine 3000 transfection kit. Viral supernatant was collected after 72 hours transfection. Then, iMEFs were cultured with viral supernatant, and the GFP‐positive cells were selected by Flow Sorting Technology. Then, we obtained three HIF‐1α KO cell lines, named as H1‐1, H1‐2, H1‐3, respectively.

### 2.6 Real‐time quantitative PCR

Total RNA was extracted using RNAiso plus (TAKARA, #1089527) and cDNA was synthesized using a prime script RT reagent kit with gDNA Eraser (TAKARA, RR047A). Real‐time quantitative polymerase chain reaction (RT‐qPCR) was performed using SYBR green™ primix Ex Taq™ Ⅱ(TAKARA, #RR820A) by Step One^®^ sequence detection system (Applied Biosystems). The relative RNA expression was calculated by using *2^−ΔΔCt^* method and the ribosomal protein GAPDH was used as a control gene to obtain normalized values. The indicated primers are shown in Table S1.

### 2.7 Immunofluorescence assay

OCT‐embedded tissues were sectioned at 6 μm thickness. The frozen slides or cells were fixed with 4.0% formaldehyde and incubated for 15 minutes. Cells were incubated with 0.1% Triton*X*‐100 for 15 minutes. Then, the cells and frozen slides were blocked in PBS containing 3% bovine serum albumin for 30 minutes at 37℃. For immunofluorescence staining, the slides and cells were incubated with primary antibodies overnight at 4℃. The slides were washed with PBS with 1% tween‐20 and incubated with the secondary immunofluorescent antibody for 1 hour in the dark. Slides were counterstained with DAPI/anti‐fade mounting medium (Vector Laboratories) and examined by the Vectra platform (Caliper Life Sciences). The primary antibodies were used as follows: Goat anti‐α‐SMA (Abcam, #ab21027), Rabbit anti‐HIF‐1α (Abcam, #ab179483), Rat anti‐Reticular Fibroblasts and Reticular Fibres antibody(ER‐TR7) (Abcam, #ab51824), Rat anti‐CD31 (BD, #550274), Rat anti‐Ki‐67 (invitrogen, #14‐5698‐80) and Rabbit anti‐PCNA (proteintech, #10205‐2‐AP).

The OCT‐embedded human samples were obtained from lung adenocarcinoma tissues and adjacent normal lung tissues, which were diagnosed with lung adenocarcinoma between May 2018 and June 2019 at the First Affiliated Hospital of Zhengzhou University. All of those patients were provided information about age, gender, pathological types and TNM stage. Each patient involved in this study was written informed consent, and the study was obtained with approval from the First Affiliated Hospital of Zhengzhou University Ethics Committee.

### 2.8 Condition medium

iMEF cells and LLC cells were cultured in regular growth medium to 70% confluence. Then, cells were washed twice with PBS and cultured with fresh DMEM for 3 days. After that, the CM was centrifuged at 12,000 *g* for 5 minutes to remove cell debris and stored at −80℃.

### 2.9 Animal experiments

C57BL/6J wild‐type mice (Female, 6‐8 weeks old) were bought from Vital River Laboratory Animal Technology Company. For xenograft experiments, LLC cells (2 × 10^5^) and iMEF cells (8 × 10^5^) were co‐injected into the mouse abdomen subcutaneously. Six days after injection, the tumour growth and weight were monitored every two days and the tumour volume was calculated as length × width × width/2. Eighteen days after injection, the mice were killed and the tumour tissues were embedded in OCT.

### 2.10 Measurement of cell viability

Cells were cultured into 96‐well plates and placed in the IncuCyte live‐cell imaging system (Essen Bio Science). Cells were observed using phase microscopy with 10× objective (NiKon, #MRH00101) and calculated by IncuCyte analysis software. In addition, 50 cells were cultured into 6‐well plates and cultured for 7 days. Then the cell clones were fixed with 4.0% formaldehyde for 15 minutes and stained with 0.1% purple crystal. The results of cell clones were detected by ChemiDoc MP Imaging System (Bio‐Rad).

### 2.11 Measurement of secreted cytokine

The concentration of CCL5 in fibroblasts CM were measured using a RANTES Mouse ELISA Kit (Invitrogen, KMC1031) according to the manufacturer's instructions.

### 2.12 Data analysis

Data analysis was performed using the SPSS software version 17.0. All results were presented as mean ± SD. The significance of difference was assessed by *t* tests or variance analysis. The *P* values < 0.05, .01 and 0.001 were considered statistically significant.

## 3 RESULTS

### 3.1 HIF‐1α is highly expressed in CAFs of LC

**FIGURE 1:**
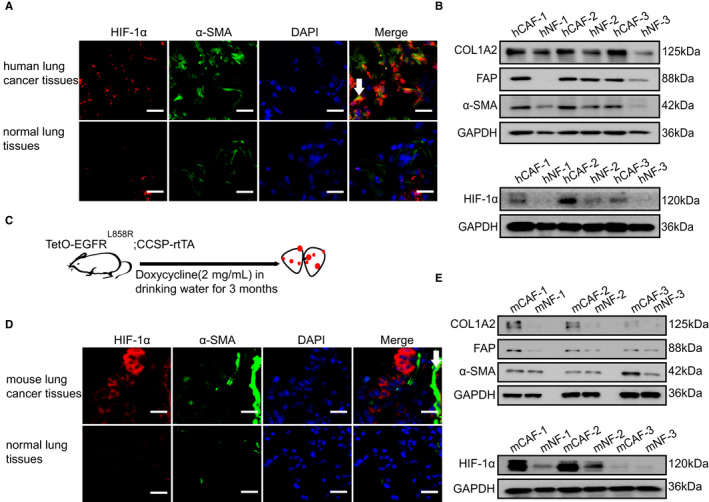
HIF‐1α is highly expressed in CAFs of LC. A, Representative immunofluorescence images of HIF‐1α (red) and α‐SMA (green) in the frozen sections from human lung cancer tissues and normal lung tissues (Scale bar, 50 μm). B, The protein levels of COL1A2, FAP, α‐SMA and HIF‐1α were determined by western blot from hCAFs and their counterpart hNFs isolated from three lung cancer patients. C, The doxycycline‐induced mouse spontaneous lung cancer model (*TetO‐EGFR^L858R^; CCSP‐rtTA*) was established. D, Representative immunofluorescence images of HIF‐1α (red) and α‐SMA (green) in the frozen sections from mouse lung cancer tissues and normal lung tissues (Scale bar, 50 μm). E, The protein levels of COL1A2, FAP, α‐SMA and HIF‐1α were determined by western blot from mCAFs and mNFs isolated from mouse lung cancer tissues and lung normal tissues

HIF‐1α is a transcription factor that is highly expressed in LC cells.^21^ However, the role of HIF‐1α in LC CAFs has not been fully investigated. To explore the relationship between the expression of HIF‐1α and CAFs in LC, the expression level of HIF‐1α was evaluated in the primary lung CAFs and NFs from LC patients. α‐SMA is an activation marker of fibroblasts in the tumour microenvironment and inflammation environment, which usually reflects the location and activity of CAFs in cancer.^3^ As shown in our results, the immunofluorescence analysis revealed that the fluorescence intensity of HIF‐1α and α‐SMA in human LC tissue was significantly higher than that in normal lung tissues (Figure 1A). Next, to further study the expression of HIF‐1α in CAFs, human CAFs (hCAFs) and NFs (hNFs) were isolated from lung tissues of LC patients and normal subjects according to the previously published protocol.^19^ For the identification of the purity in the isolated hCAFs and hNFs, we detected the expression of well‐recognized markers including α‐SMA, COL1A2 and FAP, and as expected, were at higher levels in CAFs. Meanwhile, HIF‐1α was up‐regulated in hCAFs compared with hNFs (Figure 1B). To eliminate individual human differences, a doxycycline‐induced spontaneous LC model (*TetO‐EGFR^L858R^; CCSP‐rtTA*) was established (Figure 1C). Similar to human tissues, the co‐staining intensity of α‐SMA and HIF‐1α in mouse LC tissues was higher than that in normal lung tissues (Figure 1D). As α‐SMA was a non‐specific marker of fibroblasts, we then co‐stained HIF‐1α, α‐SMA with ER‐TR7 and found that HIF‐1α is highly expressed on CAFs (Figure S1A,D). We also isolated mouse CAFs (mCAFs) and mNFs (mNFs) from LC and normal lung tissues. We found that α‐SMA, COL1A2, FAP and HIF‐1α were highly expressed in mCAFs, which was consistent with the results observed in hCAFs and hNFs (Figure 1E). To avoid the degradation of HIF‐1α, we treated CAFs and NFs with MG‐132 and found that HIF‐1α was highly expressed in hCAFs and mCAFs (Figure S1B,E). We also detected the gene expression of HIF‐1α in mCAFs and mNFs. Compared with NFs, the gene expressions of Hif1a were higher in CAFs of human lung cancer tissues and mouse lung cancer tissues (Figure S1C,F). These findings support the notion that HIF‐1α is highly expressed in lung CAFs.

### 3.2 Up‐regulation of HIF‐1α induces the transformation of fibroblasts into CAFs

**FIGURE 2:**
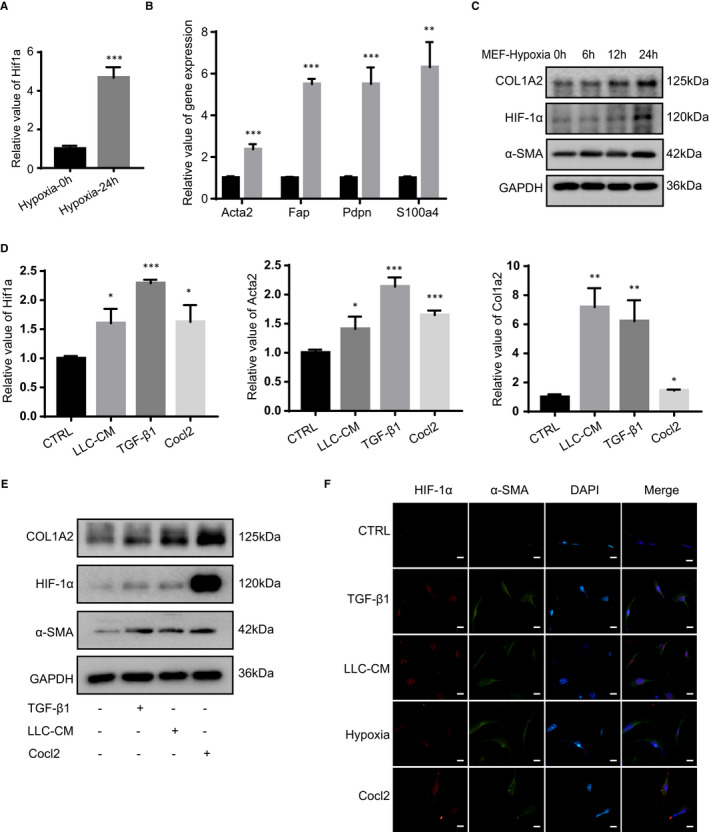
Up‐regulation of HIF‐1α induces the transformation of fibroblasts into CAFs. A, The mRNA levels of Hif1a was determined by real‐time PCR in MEF cells which were cultured under hypoxia (1% O_2_) (n = 3). B, The mRNA levels of *Acta2,* *Fap, Pdpn* and *S100a4* were determined by real‐time PCR in MEF cells which were cultured under hypoxia (n = 3). C, The protein levels of COL1A2, α‐SMA and HIF‐1α were determined by western blot in MEF cells which were cultured under hypoxia for 6, 12 and 24 h. D, The mRNA and protein levels of Hif1a, *Acta2* and Col1a2 were determined by real‐time PCR in MEF cells which were treated with LLC‐CM (the condition medium of LLC), TGF‐*β*1 (5 ng/mL) and Cocl2 (100 μmol/L) (n = 3). E, The protein levels of HIF‐1α, COL1A2 and α‐SMA were determined by western blot in MEF cells which were treated with LLC‐CM, TGF‐*β*1 and Cocl2. F, Representative immunofluorescence images of HIF‐1α (red) and α‐SMA (green) in MEF cells which were treated with LLC‐CM, TGF‐*β*1, hypoxia and Cocl2 (Scale bar, 100 μm). (*P < 0.05, **P < 0.01, ***P < 0.001)

It is well documented that hypoxia up‐regulates the expression of HIF‐1α.^13^ To gain further insights into the effects of HIF‐1α on fibroblasts, primary MEF cells were isolated from C57 mouse embryo and treated with hypoxia. After 24 hours of treatment, a significant up‐regulation of Hif1a was observed (about 5‐fold) (Figure 2A). Simultaneously, hypoxia drastically facilitated the gene expression of Acta2, Pdpn, Fap and S100a4 (Figure 2B), which were reported as CAFs markers.^3^ After the treatment of hypoxia in MEF cells, we also found HIF‐1α, α‐SMA and COL1A2 increased in a time‐dependent manner at the protein levels (Figure 2C). These results suggest that hypoxia can up‐regulate the expression of HIF‐1α and induce fibroblasts to transform into CAFs.

Cocl_2_ is used to mimic hypoxia to induce the expression of HIF‐1α.^22^ TGF‐*β*1 and tumour cell CM are reported to activate the fibroblasts and increase the deposition of ECM.^8^ To investigate the expression of HIF‐1α during the activation of fibroblasts, MEF cells were treated with three kinds of stimulating factors (TGF‐*β*1 and tumour cell CM, as well as Cocl_2_). First, we confirmed that Hif1a expression was up‐regulated at the RNA level (Figure 2D). At the same time, we also detected the expression of HIF‐1α at the protein level and found that the expression of HIF‐1α was increased under the stimulators (Figure 2E). To further determine the effect of HIF‐1α expression on the activation of fibroblasts, we examined the effects of TGF‐*β*1, tumour cell CM and Cocl_2_ on Acta2 and Col1a2, which are markers of CAFs.^9^ As shown in the figure, TGF‐*β*1, tumour cell CM and Cocl_2_ notably increased the expression of Col1a2 and Acta2 of fibroblasts at RNA levels (Figure 2D). Moreover, the expression of α‐SMA and COL1A2 was up‐regulated, consisted with HIF‐1α at protein levels (Figure 2E). Subsequently, under the treatment of TGF‐*β*1, tumour cell CM, hypoxia and Cocl_2_, HIF‐1α and α‐SMA expression were detected by immunofluorescence in fibroblasts, which showed significant up‐regulated fluorescence intensities (Figure 2F). To sum up, consistent with the effect of TGF‐*β1* and tumour cell CM, Cocl_2_ and hypoxia promote the expression of HIF‐1α and induced the transformation of fibroblasts into CAFs.

### 3.3 HIF‐1α is essential for the activation of fibroblasts

**FIGURE 3:**
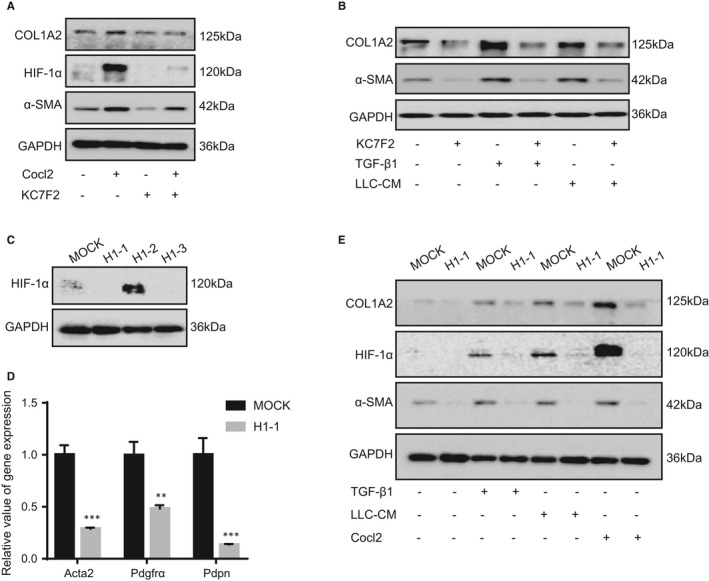
HIF‐1α is essential for the activation of fibroblasts. A, The protein levels of HIF‐1α, COL1A2 and α‐SMA were detected by western blot in MEF cells which were treated with Cocl2 and HIF‐1α inhibitor KC7F2 (10 μmol/L). B, The protein levels of COL1A2 and α‐SMA were determined by western blot in MEF cells which were treated with KC7F2, TGF‐*β*1 and LLC‐CM. C, The protein levels of HIF‐1α was stably knocked out (KO) by CRISPR‐*Cas9* in iMEF cells. D, The mRNA levels of *Acta2*, *Pdgfr*α and *Pdpn* were determined by real‐time PCR in HIF‐1α MOCK (MOCK) cells and HIF‐1α KO (H1‐1) cells (n = 3). E, The protein levels of COL1A2, α‐SMA and HIF‐1α were determined by western blot in MOCK and H1‐1 cells which were treated with TGF‐*β*1, LLC‐CM and Cocl2. (**P < 0.01, ***P < 0.001)

In order to determine the relationship between HIF‐1α and the activation of fibroblasts, we conducted a series of experiments in vitro. First, the MEF cells were treated with Cocl_2_ and HIF‐1α inhibitor (KC7F2) to investigate the effect of HIF‐1α on the activation of fibroblasts.^23^ As shown in Figure 3A, fibroblasts treated with Cocl_2_ exhibited a promotion effect on the expression of HIF‐1α, α‐SMA and COL1A2. However, KC7F2 neutralized the active status of MEF cells and decreased the expression of HIF‐1α induced by Cocl2 (Figure 3A). In addition, KC7F2 also could attenuate the effect of TGF‐*β*1 and tumour cell CM on the expression of α‐SMA and COL1A2 (Figure 3B).

Furthermore, it was also observed that HIF‐1α KO in fibroblasts restrained the gene expression of Acta2, Pdgfrα and Pdpn (Figure 3C,D). Then, the HIF‐1α *MOCK* fibroblasts and HIF‐1α KO fibroblasts were treated with TGF‐*β1*, tumour cell CM and Cocl2, respectively. The expressions of HIF‐1α, α‐SMA and COL1A2 were increased in HIF‐1α MOCK fibroblasts, but did not alter in HIF‐1α KO fibroblasts, suggesting that HIF‐1α was involved in the activation of fibroblasts (Figure 3E). We also detected the gene expression of Epas1 in HIF‐1α MOCK and HIF‐1α KO cells and found that the expression of Epas1 is decreased in HIF‐1α KO cells (Figure S2). Overall, these data indicate that HIF‐1α is necessary to mediate the activation of fibroblasts.

### 3.4 Knockout of HIF‐1α in fibroblasts attenuates the growth of LC

**FIGURE 4:**
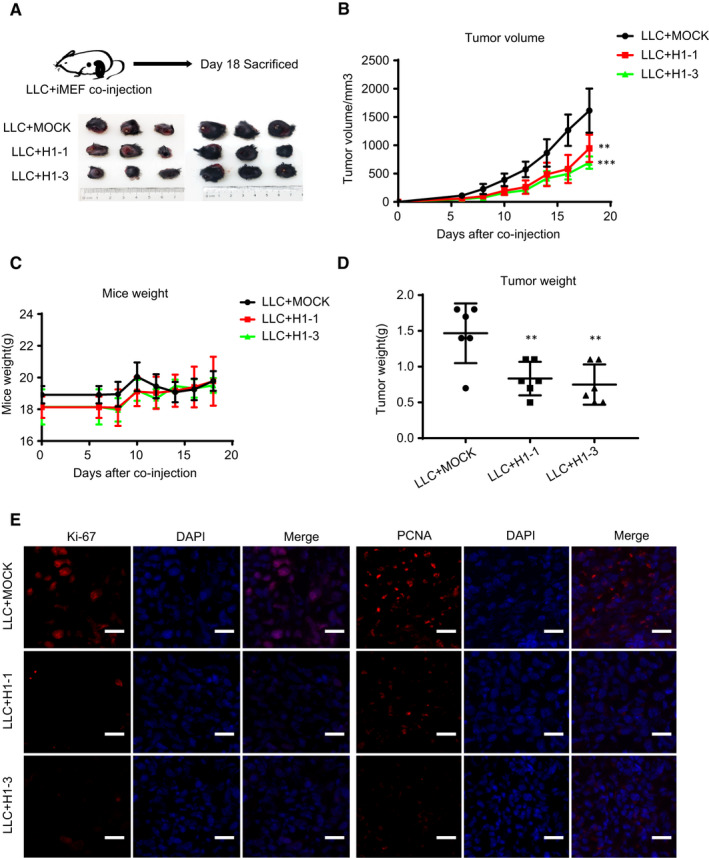
Knockout of HIF‐1α in fibroblasts attenuates the growth of LC. A, The HIF‐1α MOCK cells or HIF‐1α KO cells and LLC cells were subcutaneously co‐injected into the C57 mice (n = 6 mice/group) and the representative tumour xenograft images were exhibited. B and C, The tumour xenograft volumes and mouse weights were measured every 2 d. D, The tumour xenograft weights were measured after sacrifice. E, Representative immunofluorescence images of Ki‐67 (red) and PCNA (red) in the frozen sections from the tumour xenograft tissues (Scale bar, 50 μm). (**P < 0.01, ***P < 0.001)

Cancer‐associated fibroblasts are known to have an important role in tumour growth.^4^ To identify whether fibroblasts with HIF‐1α expression contribute to the promotion of tumour growth in vivo, we co‐injected HIF‐1α MOCK fibroblasts or HIF‐1α KO fibroblasts with LLC cells into the C57 mice to detect the growth of lung tumour (Figure 4A). As shown in our data, the tumour formation and growth in HIF‐1α KO fibroblasts‐injected group were significantly lower than that in HIF‐1α MOCK fibroblasts‐injected one, and the tumour volume and weight in the experimental mice were significantly smaller than those in the control group, while the mice weights had no obvious difference between two groups (Figure 4B‐D). Interestingly, the expression of CD31 in HIF‐1α KO fibroblasts tumours is similar to that in HIF‐1α MOCK fibroblasts (Figure S3). PCNA and Ki‐67 are the markers of cell proliferation. To explore the mechanism of the HIF‐1α in fibroblasts promoting tumour growth, PCNA and Ki‐67 immunofluorescence analysis were used. We found that PCNA and Ki‐67 were abundant in HIF‐1α MOCK fibroblasts tumour tissues (Figure 4E). Those results show that the loss of HIF‐1α in fibroblasts restrains the lung tumour growth by suppressing the tumour cell proliferation.

### 3.5 HIF‐1α‐expressed fibroblasts secrete CCL5 to drive the growth of LC

**FIGURE 5:**
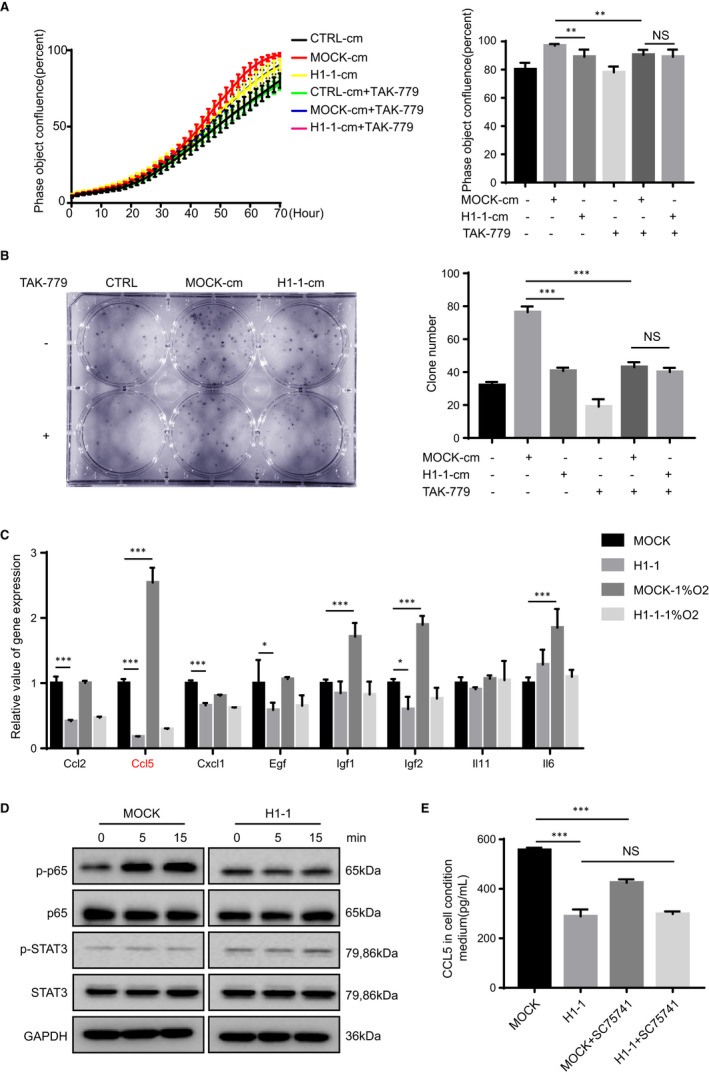
HIF‐1α‐expressed fibroblasts secrete *CCL5* to drive the growth of LC. A, By treatment of CM of MOCK cells and H1‐1 cells, the cell proliferation abilities of LLC cells were observed and measured. CCL5 receptor inhibitor TAK‐779 (10 nmol/L) was added in the condition mediums. The right column is the statistics of cell proliferation abilities at 70 h (n = 3). B, By treatment of CM of MOCK cells and H1‐1 cells, the cell clones of LLC were observed and measured. CCL5 receptor inhibitor TAK‐779 was added in the condition mediums (n = 3). C, The mRNA levels of *Ccl2, Ccl5, Cxcl1,* *Egf, Igf1, Igf2, Il11* and *Il6* were determined by real‐time PCR in the MOCK cells and H1‐1 cells treated with or without hypoxia (1% O_2_) (n = 3). D, The protein *p‐p65, p65*, *p*‐STAT3 and STAT3 were determined by western blot in MOCK cells and H1‐1 cells which were treated with Cocl2 for 5 and 15 min. E, The secretion levels of CCL5 in the CM of MOCK cells and H1‐1 cells were analyzed by ELISA kit. *NF‐kB* inhibitor SC75741 (1 μmol/L) was added to inhibit the cellular *NF‐kB* signaling pathway (n = 3). (*P < 0.05, **P < 0.01, ***P < 0.001)

To determine whether the HIF‐1α‐expressed fibroblasts contribute to tumour promotion, tumour cells were treated with fibroblasts CM. The data demonstrated that compared with HIF‐1α KO fibroblasts, HIF‐1α MOCK fibroblasts enhanced tumour cell proliferation and phenotype cloning (Figure 5A,B). CAFs are known to secrete multiple of pro‐inflammatory cytokines and chemokines during tumour progression.^3^ To detect the difference between HIF‐1α MOCK fibroblasts and HIF‐1α KO fibroblasts, we measured the expression levels of 8 genes (Ccl2, Ccl5, Cxcl1, Egf, Igf1, Igf2, Il11, Il6) secreted by CAFs. As shown in Figure 5C, we found that the RNA levels of Ccl5, Ccl2, Cxcl1, Egf and Igf2 were substantially up‐regulated in the HIF‐1α MOCK fibroblasts, but many growth factors like Igf1, Il11 and Il6 remained unchanged. To avoid the possible off‐target effects associated with HIF‐1α KO fibroblasts, we treated HIF‐1α MOCK fibroblasts and HIF‐1α KO fibroblasts with hypoxia and found that Ccl5, Igf1, Igf2 and Il6 were increased significantly in HIF‐1α MOCK fibroblast. Meanwhile, the expression of those factors in HIF‐1α KO fibroblast has no changes. As the expression of Ccl5 was mostly reduced (about 5‐folds) in HIF‐1α KO fibroblasts and up‐regulated (about 2.5‐folds) in HIF‐1α MOCK fibroblasts treated with hypoxia (Figure 5C), we speculated that CCL5 might play important roles in the promotion of tumour cell growth. We then detected the expression of CCR5 in LLC, MEF, A549 and MRC‐5 cells. Our data showed that CCR5 was highly expressed in LLC and A549 LC cells (Figure S4A). TCGA database showed that the expression of CCR5 was significantly increased in human LC (Figure S4B). Next, ELISA analyses showed that HIF‐1α MOCK fibroblasts secreted more CCL5 than HIF‐1α KO fibroblasts (Figure 5E), indicating that CCL5 might be the main factor in the tumour promoting effect of HIF‐1α MOCK fibroblasts. TAK‐779 is an inhibitor of CCR5, which could inhibit the effect of CCL5 on tumour cells.^24^ As a result, TAK‐779 neutralized the enhancement effect of HIF‐1α expression fibroblasts on the proliferation and clone formation of tumour cells (Figure 5A,B).

CCL5 is a well‐known target of nuclear factor‐*κB* (*NF‐κB*) and STAT3 signalling pathways.^25^, ^26^ Then, we determined the levels of *p65, p‐p65,* STAT3 and *p‐*STAT3 in our experiment model. Compared with HIF‐1α KO fibroblasts, Cocl_2_ increased *p‐p65* expression in HIF‐1α MOCK fibroblasts. However, the expression of *p‐*STAT3 and STAT3 had no difference between HIF‐1α MOCK fibroblasts and HIF‐1α KO fibroblasts under the stimulation of Cocl_2_ (Figure 5D). Furthermore, SC75741, a *p‐p65* inhibitor also inhibited the secretion of CCL5 in HIF‐1α MOCK fibroblasts, rather than HIF‐1α KO fibroblasts (Figure 5E). Those results suggested that the HIF‐1α‐expressed fibroblasts secreted CCL5 via *NF‐κB* signalling pathway, which further promotes the tumour growth.

**FIGURE 6:**
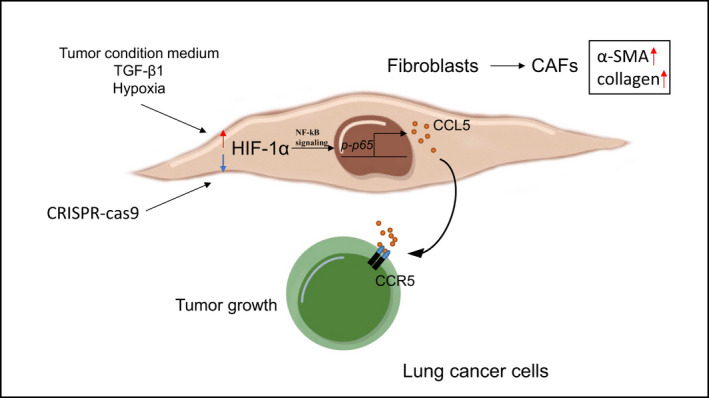
The schematic diagram for the HIF‐1α promoting fibroblasts activation to drive the growth of LC. We propose a schematic diagram for the HIF‐1α promoting fibroblast activation to drive the growth of LC. First, the tumour CM, TGF‐*β*1 and hypoxia up‐regulate the HIF‐1α expression in fibroblasts, inducing the conversion of fibroblasts into CAFs. Second, the fibroblasts with high HIF‐1α expression secrete CCL5 by activating *NF‐kB* signaling pathway, thus promoting the tumour growth of LC

In conclusion, our data indicated that HIF‐1α was highly expressed in lung CAFs. Additionally, hypoxia, TGF‐*β*1 and tumour cell CM up‐regulated the expression of HIF‐1α in fibroblasts, inducing the conversion of fibroblasts into CAFs. Moreover, HIF‐1α‐expressed CAFs secreted CCL5 by activating *NF‐κB* signalling pathway, thus promoting the tumour growth of LC (Figure 6).

## 4 DISCUSSION

Recently, many genetic engineering models have been developed to study LC. Several driver oncogenes are identified in human genome research, such as *k‐ras*, *EGFR* mutant, which have been altered to induce LC models.^27^ In this study, *TetO‐EGFR^L858R^; CCSP‐rtTA* genetic engineering model was established by using doxycycline‐containing diets as materials.^19^ This model is a spontaneous lung tumour model, which can simulate the process of LC to the maximum extent. So far, few studies have applied *TetO‐EGFR^L858R^; CCSP‐rtTA* model to investigate CAFs in LC. In the process of the tumour growth, CAFs are associated with the prognosis of many cancers.^4^ However, little was known about the exact role of CAFs in LC. The aim of the present study was to evaluate the expression of HIF‐1α in CAFs. In the previous study, α‐SMA is used to mark the fibroblast.^28^, ^29^, ^30^ Furthermore, α‐SMA is significantly up‐regulated after fibroblast activation and used as a surface marker for fibroblast subset.^31^ So we co‐stained the HIF‐1α and α‐SMA in lung cancer tissues and normal lung tissues, our results demonstrated that HIF‐1α was highly expressed in CAFs of human LC tissues and mouse spontaneous LC tissues. But some researchers point that α‐SMA is also expressed by vascular pericytes in tumours.^32^ ER‐TR7 is expressed in the cytoplasm of fibroblasts and connective tissue, and used to label fibroblasts in our experiments.^33^ Both α‐SMA and ER‐TR7 positive cells were defined as the CAFs. Most importantly, the morphology and distribution of fibroblasts in the tissues can be used to observe the expression of HIF‐1α in the fibroblast of lung cancer tissues and normal lung tissues. In addition, we also isolated CAFs and NFs from lung cancer tissues and normal lung tissues, revealing that the expression of HIF‐1α was highly expressed in CAFs. Studies have shown that the Von Hippel‐Lindau (VHL) tumour suppressor protein can bind prolyl‐hydroxylated HIF‐1α and promote its proteasomal degradation.^34^ To eliminate the degradation of HIF‐1α, we treated CAFs and NFs with MG‐132, the inhibitor of proteasome, and found that HIF‐1α was indeed highly expressed in CAFs. Researchers found that three types of CAFs including myCAFs, iCAFs and apCAFs had been identified in pancreatic ductal adenocarcinoma. Among them, HIF‐1α is significantly up‐regulated in iCAFs, which is consistent with our data.^35^ As the gene regulation is higher in our results and the proteasome degradation could not change the high expression of HIF‐1α in CAFs, which results the expression of HIF‐1α in CAFs is higher. However, it is unclear whether there are upstream genes in CAFs that regulate the expression of HIF‐1α, it needs to further investigate for us.

Hypoxia is the main characteristic of LC microenvironment and also remodels the composition of TME through inadequate oxygen availability.^15^, ^36^ Besides hypoxia, Cocl_2_ is often used as a chemical inducer of hypoxia by enhancing the expression of HIF‐1α to mimic hypoxia.^37^ In our results, hypoxia and Cocl_2_ up‐regulated HIF‐1α, and thereby enhanced the expression of fibroblast activation markers such as *a*‐SMA and COL1A2. However, the relationship between HIF‐1α expression and fibroblasts activation is not clear in LC. CRISPR‐cas9 was used to knock out the expression of HIF‐1α. Due to the off‐target effect of CRISPR‐cas9 technology, the upstream molecules which regulate the expression of HIF‐1α may be targeted. That's why we choose H1‐1 to conduct the following experiments. HIF is a heterodimer consisting of three alpha subunits (HIF‐1α, HIF‐2α, and HIF‐3α).^38^ Under hypoxic conditions, both HIF‐1α and HIF‐2α are stabilized and the main function of HIF‐2α is involved in regulating EPO and IRS2 in liver.^39^ As shown in the results, when the expression of HIF‐1α was knocked out, the expression of HIF‐2α was decreased. Therefore, HIF‐2α can't compensate the loss of HIF‐1α in fibroblasts.

Fibroblasts are activated under the stimulation of tumour microenvironment, including TGF‐*β1*, PDGF, SHH, BMP, IL‐1, IL‐6 and tumour necrosis factor (TNF).^3^, ^5^, ^7^ The activation of fibroblasts in TME is not only by cytokines and the physical factors, but also by the changes of cells themselves. Among these, TGF*‐β*1 signalling activates the resident fibroblasts to increase the metastatic potential and chemotherapy resistance of tumour cells.^40^, ^41^ Researchers also found that all subtypes of CRC with poor prognosis have common genetic programs induced by TGF‐β1 in tumour stromal cells.^42^ In addition to TGF‐*β*1, tumour cell CM is commonly used to stimulate the fibroblasts.^43^ Our study suggested that the expression of HIF‐1α was affected by hypoxia, Cocl_2_, LLC‐CM and TGF‐*β*1. Based on the above results, hypoxia‐induced high expression of HIF‐1α might represent the characteristics of CAFs.

The effect of HIF‐1α in fibroblasts on LC cells is not known yet. Recent studies show that the loss of HIF‐1αin FAP positive fibroblasts can reduce the vascular density and myeloid cell infiltration to promote the tumour perfusion in breast cancer model.^15^ On the contrary, our results demonstrated that the loss of HIF‐1α in fibroblasts decreased the tumour growth, while the vascular density had no significant difference between the HIF‐1α KO fibroblasts and HIF‐1α MOCK fibroblasts. We assume that the conflicting results may be due to the following reasons. Firstly, HIF‐1α in fibroblasts has both pro‐tumour and anti‐tumour effects according to the type and malignant degree of cancer. Secondly, the role of FAP may be contrary to that of loss of HIF‐1α, leading to the promotion of tumour growth. CAFs play a tumour promoting role in TME by secreting immunosuppressive cytokines. For instance, CAFs secrete growth factors such as CXCL12, HGF, EGF, IGF, IL‐6, IL‐8, IL‐11 and CCL5, which facilitate tumour growth.^3^ Our results displayed that the growth factor in fibroblast CM induced the rapid proliferation of LC cells, and the expression of CCL2 was reduced in HIF‐1α KO fibroblasts. However, other researchers have found that the role of CCL2 in TME is to recruit MDSC and macrophages, thus providing an immunosuppressive microenvironment.^44^ As the off‐target effect of CRISPER‐cas9, we treated HIF‐1α MOCK fibroblasts and HIF‐1α KO fibroblasts with hypoxia and found that the Ccl5, Igf1, Igf2 and Il6 were up‐regulated. Studies have shown that HIF‐1α is not only response to hypoxia, but also can be regulated by oncogene activation or tumour suppressor gene inactivation.^45^ We speculate that Igf1, Igf2 and Il6 may be up‐regulated through other pathways after hypoxia stimulation, and that they are not the targets of HIF‐1α. However, Ccl5 was greatly reduced in HIF‐1α KO fibroblasts and mostly up‐regulated in HIF‐1α MOCK fibroblasts treated with hypoxia. Therefore, Ccl5 might be the target of HIF‐1α in fibroblasts and was selected in the following experiment.

CCL5 mediates diverse biological functions and its main receptor is C‐C chemokine receptor type 5 (CCR5).^46^ The secretion of CCL5 mainly comes from T lymphocytes, macrophages, platelets, fibroblasts and several types of tumour cells, which is not only related to the immune response against tumours, but also to the cancer progression and metastasis.^47^, ^48^ In ovarian cancer, the increased *miR‐155* expression can transform fibroblasts into CAFs, thereby increasing the production of CCL5 and promoting the growth of tumour cells.^49^ We found that the expression of CCR5 was highly expressed in LC cells. The secretion of CCL5 from HIF‐1α high expressed fibroblasts might be combined with CCR5 to promote the growth of tumour cells. Studies have also shown that CCL5 can promote the growth of colorectal cancer and prostate cancer, which can be inhibited by TAK‐779, a CCR5 antagonist.^24^, ^50^ Next, we found that CCL5 was an important factor in CAFs promoting tumour growth, and *NF‐κB* signalling pathway was involved in enhancing CCL5 secretion. When the effect of CCL5 was blocked by TAK‐779, the HIF‐1α‐expressed fibroblasts CM could not promote the growth of tumour cells. Some studies have pointed out that CCL5 is a target of *NF‐κB* and STAT3 signalling pathways.^26^ Our data suggest that the *NF‐κB* signalling pathway is indeed regulated by HIF‐1α. In addition, the STAT3 signalling pathway was inactivated in both HIF‐1α MOCK fibroblasts and HIF‐1α KO fibroblasts treated with Cocl2, indicating that HIF‐1α could not regulate STAT3 signalling pathway. We then validated that *NF‐κB* signal inhibitor suppressed the secretion of CCL5 in fibroblasts, suggesting that HIF‐1α‐expressed fibroblasts secreted CCL5 via *NF‐κB* signalling pathway. In tumour microenvironment, CCL5 is involved in many immune responses in TME.^3^ But we have not detected whether HIF‐1α‐expressed fibroblasts interact with other immune cells, such as tumour‐associated macrophages, T cells, MDSC. We speculated that there might be other mechanisms of HIF‐1α labelled CAFs to promote the growth of LC, which provides a new research direction for future studies.

In summary, this study indicates that HIF‐1α is highly expressed in CAFs, and HIF‐1α‐expressed fibroblasts secreted CCL5 by activating *NF‐κB* signalling pathway, thus promoting the tumour growth of LC. Our study identify that HIF‐1α is an essential factor of CAFs in LC and targeting HIF‐1α‐expressed CAFs has potential in future anticancer therapy.

## CONFLICTS OF INTEREST

The authors declare no conflict of interest.

## AUTHOR CONTRIBUTIONS

**Yana Zhang:** Formal analysis (lead); Investigation (lead); Methodology (lead); Software (lead); Visualization (lead); Writing‐original draft (lead); Writing‐review & editing (equal). **Yangyang Bian:** Conceptualization (supporting); Project administration (supporting). **Yuan Wang:** Methodology (supporting). **Yuanyuan Wang:** Conceptualization (supporting); Methodology (supporting). **Xixi Duan:** Methodology (supporting). **Yuning Han:** Writing‐review & editing (supporting). **LiJing Zhang:** Methodology (supporting). **Fei Wang:** Methodology (supporting). **Zhuoyu Gu:** Project administration (supporting); Validation (equal); Visualization (supporting); Writing‐original draft (supporting); Writing‐review & editing (lead). **Zhihai Qin:** Conceptualization (equal); Funding acquisition (lead); Project administration (equal); Supervision (equal); Writing‐original draft (supporting); Writing‐review & editing (supporting).

## Supporting information

Fig S1

Fig S2

Fig S3

Fig S4

Table S1

Table S2

## Data Availability

All data supporting the findings of this study are available within the article and its Supporting Information files.
