# Supplementary figures and images for: HIF‐1α is necessary for activation and tumour‐promotion effect of cancer‐associated fibroblasts in lung cancer
Source: J Cell Mol Med. 2021 May 4;25(12):5457–69. doi: 10.1111/jcmm.16556 (PMC8184678; doi:10.1111/jcmm.16556)

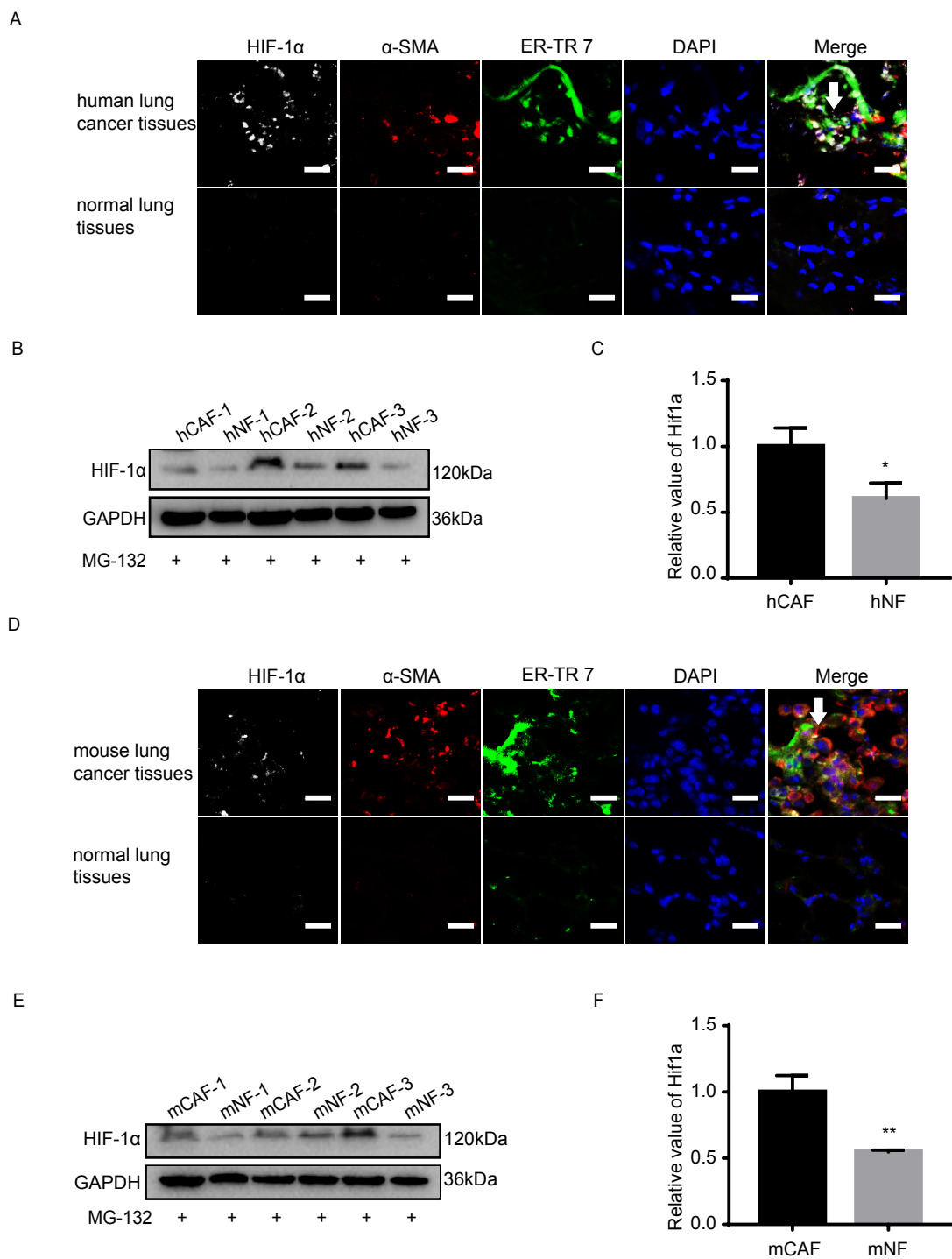

FIGURE S1

Supplement: Supplementary file 1 — Fig S1 [file JCMM-25-5457-s006.pdf]

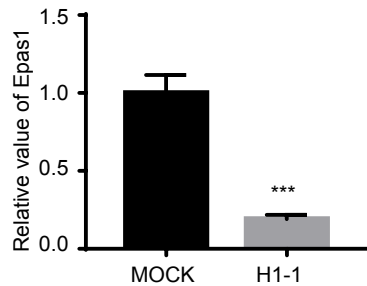

FIGURE S2

Supplement: Supplementary file 2 — Fig S2 [file JCMM-25-5457-s005.pdf]

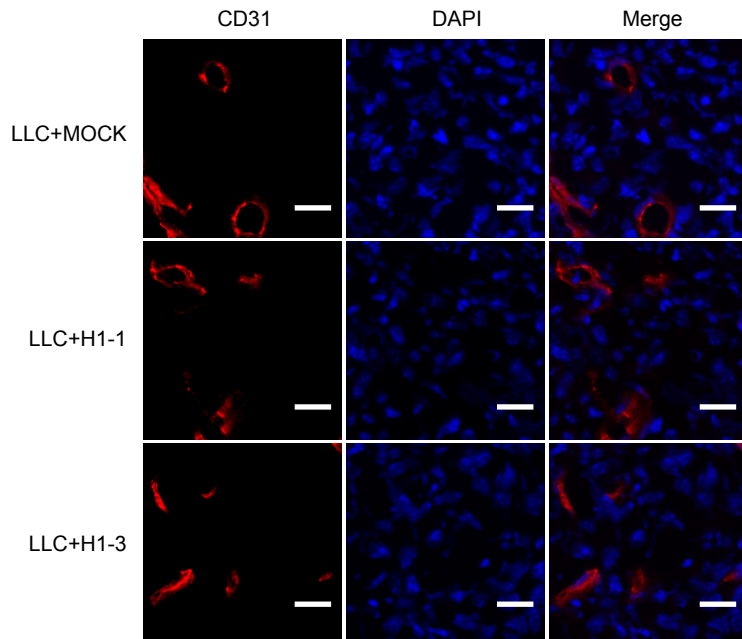

FIGURE S3

Supplement: Supplementary file 3 — Fig S3 [file JCMM-25-5457-s001.pdf]

A

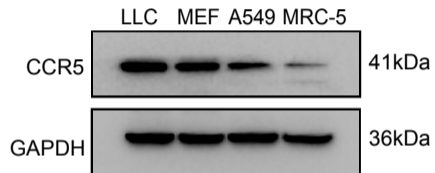

B

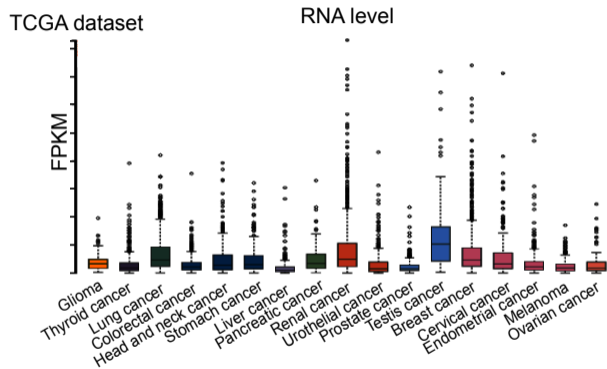

FIGURE S4

Supplement: Supplementary file 4 — Fig S4 [file JCMM-25-5457-s004.pdf]
