# Supplementary material for: HIF‐1α is necessary for activation and tumour‐promotion effect of cancer‐associated fibroblasts in lung cancer
Source: J Cell Mol Med. 2021 May 4;25(12):5457–69. doi: 10.1111/jcmm.16556 (PMC8184678; doi:10.1111/jcmm.16556)
Supplement: Supplementary file 5 — Table S1 [file JCMM-25-5457-s003.docx]

**Table S1** The primers in our study are shown as follows

| Primer sequence |  |  |
| --- | --- | --- |
| Gapdh(mouse) | Forward(5'-3') | AGGTCGGTGTGAACGGATTTG |
|  | Reword(5'-3') | GGGGTCGTTGATGGCAACA |
| S100a4(mouse) | Forward(5'-3') | TGAGCAACTTGGACAGCAACA |
|  | Reword(5'-3') | CTTCTTCCGGGGCTCCTTATC |
| Acta2(mouse) | Forward(5'-3') | CCCAGACATCAGGGAGTAATGG |
|  | Reword(5'-3') | TCTATCGGATACTTCAGCGTCA |
| Fap(mouse) | Forward(5'-3') | GTCACCTGATCGGCAATTTGT |
|  | Reword(5'-3') | TCGTAGATGTAGTATGTCGCTGT |
| Hif1a(mouse) | Forward(5'-3') | TCTCGGCGAAGCAAAGAGTC |
|  | Reword(5'-3') | AGCCATCTAGGGCTTTCAGATAA |
| Il6(mouse) | Forward(5'-3') | CTGCAAGAGACTTCCATCCAG |
|  | Reword(5'-3') | AGTGGTATAGACAGGTCTGTTGG |
| Igf1(mouse) | Forward(5'-3') | CTGGACCAGAGACCCTTTGC |
|  | Reword(5'-3') | GGACGGGGACTTCTGAGTCTT |
| Il11(mouse) | Forward(5'-3') | GCGCTGTTCTCCTAACCCG |
|  | Reword(5'-3') | GAGTCCAGACTGTGATCTCCG |
| Igf2(mouse) | Forward(5'-3') | GTGCTGCATCGCTGCTTAC |
|  | Reword(5'-3') | CGGTCCGAACAGACAAACTG |
| Egf(mouse) | Forward(5'-3') | AGAGCATCTCTCGGATTGACC |
|  | Reword(5'-3') | CCCGTTAAGGAAAACTCTTAGCA |
| Cxcl1(mouse) | Forward(5'-3') | CTGGGATTCACCTCAAGAACATC |
|  | Reword(5'-3') | CAGGGTCAAGGCAAGCCTC |
| Ccl2(mouse) | Forward(5'-3') | TTAAAAACCTGGATCGGAACCAA |
|  | Reword(5'-3') | GCATTAGCTTCAGATTTACGGGT |
| Ccl5(mouse) | Forward(5'-3') | GCTGCTTTGCCTACCTCTCC |
|  | Reword(5'-3') | TCGAGTGACAAACACGACTGC |
| Pdpn(mouse) | Forward(5'-3') | GTTTTGGGGAGCGTTTGGTTC |
|  | Reword(5'-3') | CATTAAGCCCTCCAGTAGCAC |
| Col1a2(mouse) | Forward(5'-3') | TCGTGCCTAGCAACATGCC |
|  | Reword(5'-3') | TTTGTCAGAATACTGAGCAGCAA |
| Pdgfrα(mouse) | Forward(5'-3') | TATCCTCCCAAACGAGAATGAGA |
|  | Reword(5'-3') | GTGGTTGTAGTAGCAAGTGTACC |
| EPAS1(mouse) | Forward(5'-3') | GAGGAAGGAGAAATCCCGTGA |
|  | Reword(5'-3') | TATGTGTCCGAAGGAAGCTGA |
