# Supplementary material for: HIF‐1α is necessary for activation and tumour‐promotion effect of cancer‐associated fibroblasts in lung cancer
Source: J Cell Mol Med. 2021 May 4;25(12):5457–69. doi: 10.1111/jcmm.16556 (PMC8184678; doi:10.1111/jcmm.16556)
Supplement: Supplementary file 6 — Table S2 [file JCMM-25-5457-s002.docx]

**Table S2**

| Abbreviation | Full name |
| --- | --- |
| CAFs | Cancer associated fibroblasts |
| LC | Lung cancer |
| TME | Tumour microenvironment |
| NFs | Normal fibroblasts |
| KO | Knockout |
| α-SMA/ACTA2 | α-smooth muscle actin |
| HIF-1*α* | Hypoxia inducible factor-1*α* |
| FAP | Fibroblast-activating protein |
| PDGFR*α* | Platelet-derived growth factor receptor-*α* |
| S100A4/FSP1 | Fibroblast-specific protein 1 |
| PDPN | Podoplanin |
| NSCLC | Non-small cell lung cancer |
| PDAC | Pancreatic ductal adenocarcinoma |
| PCNA | Proliferating cell nuclear antigen |
| CM | Condition medium |
| LLC | Lewis lung cancer cells |
| mCAFs | Mouse cancer associated fibroblasts |
| mNFs | Mouse normal fibroblasts |
| HRP | Horseradish-peroxidase-conjugated |
| MEF | Mouse embryonic fibroblast |
| iMEF | Immortalized mouse embryonic fibroblast |
| DMEM | Dulbecco’s modified eagle medium |
| hCAFs | Human cancer associated fibroblasts |
| hNFs | Human normal fibroblasts |
| Cocl2 | Cobalt chloride |
| PDGFR*β* | Platelet-derived growth factor receptor-*β* |
| PDGF | Platelet-derived growth factor |
| SHH | Sonic hedgehog |
| TNF | Tumour necrosis factor |
| BMP | Bone morphogenetic protein |
| CCR5 | C-C chemokine receptor type 5 |
